# A Tumour Cell Aggregation Promoting Substance from Rat Ascites Hepatoma Cells*

**DOI:** 10.1038/bjc.1974.234

**Published:** 1974-12

**Authors:** K. Kudo, I. Tasaki, Y. Hanaoka, H. Hayashi

## Abstract

**Images:**


*Br. J. Cancer* (1974) **30**, 549

### A TUMOUR CELL AGGREGATION PROMOTING SUBSTANCE FROM RAT ASCITES HEPATOMA CELLS*

K. KUDO, I. TASAKI, Y. HANAOKA AND H. HAYASHI

*From the Department of Pathology, Kumamoto University Medical School, Kumamoto 860, Japan*

Received 28 June 1974. Accepted 15 July 1974

**Summary.—A substance capable of promoting tumour cell aggregation was released from rat ascites hepatoma cell (possibly from the cell surface) kept in Hanks' balanced salt solution (free of calcium and magnesium) in the cold, and then partially purified by chromatography with DEAE-Sephadex and gel filtration with Bio-gel. The thermostable substance seemed to be a glycoprotein and its molecular weight was about 72,000 when measured by gel filtration on Sephadex G-200. It had no proteolytic activity. The material was clearly effective for rat ascites hepatoma cells as well as SV40 transformed cells, but less effective for Chang's cells and apparently ineffective for normal rat liver cells and red blood cells. The action of this material was more potent than that of Jack bean concanavalin A when assayed for aggregation of SV40 transformed cells. Its effect was not influenced by concanavalin A inhibitors such as alpha-methyl-D-glucopyranoside, N-acetyl-D-glucosamine and D-glucose.**

THE MECHANISMS of invasion in cancer have not yet been established. Various explanations have been given for these mechanisms: (a) a decrease in mutual adhesiveness of tumour cells, by which the cells become free from each other (Coman, 1944; Zeidman, 1947; McCutheon, Coman and Moore, 1948); (b) an increase in ameboid motility and loss of contact inhibition of tumour cells, by which the cells invade the interstitial spaces of the adjacent normal tissues (Hanes and Lambert, 1912; Enterline and Coman, 1950; Hirono, 1958; Abercrombie and Ambrose, 1962; Wood, Robinson and Marzocchi, 1968); (c) invasion by tumour cells of small vessels, by means of which the cells are transported to distant organs (Sato, Suzuki and Kurokawa, 1966); and (d) migration of tumour cells through the vessel wall whereby, under favourable conditions, they lodge and proliferate to form metastatic secondary tumours (Nakamura, 1964; Satoh, 1967). It has been suggested that the invasive character of cancer cells may be in some way associated with an increased locomotion of the cells (Hirono, 1958; Sato, 1967).

As described in previous papers (Hayashi *et al.*, 1970; Yoshida *et al.*, 1970), a substance chemotactic for cancer cells has been isolated from some tumour tissues of animal and human origin. After injection, this substance locally induced an extravascular migration of circulating tumour cells and the formation of metastatic tumour (Ozaki *et al.*, 1971). Since the chemotactic factor could only be isolated from tumour tissues but not from the cancer cells themselves, it was supposed that the chemotactic factor was produced outside the cancer cells. It was also demonstrated that local production of the chemotactic factor were associated with the action of a certain neutral protease from cancer cells, *e.g.* rat ascites hepatoma cells (Koono, Ushijima and Hayashi, 1974) and that activation and release of the neutral protease were associated with a certain thermostable peptide

* This is No. 1 of the studies on tumour cell aggregation promoting factor.

from tumour tissues (Koono, Katsuya and Hayashi, 1974). In a further study (Katsuya, Koono and Hayashi, 1973), it was suggested that dissociation of rat ascites hepatoma cells was related to the action of the neutral protease activated by the peptide.

On the other hand, mechanisms which control cell adhesiveness have been suggested to be intimately related to the surface properties of tumour cells. The introduction of the rotational method of promoting cell aggregation led to the possibility of obtaining reproducible data (Moscona, 1961*a*). The aggregation of dissociated cells from sponges (Humphreys 1963), chick and mouse embryos (Moscona, 1961*b*), and tissue culture cells (Moskowitz, 1963) had been investigated using this method, and it has been suggested that specific macromolecular constituents of the cell surface might be involved in the phenomenon (Lilien, 1698). The mechanism of tumour cell adhesiveness is undoubtedly important in the explanation of malignant invasion. The purpose of the present communication is to describe the isolation of an aggregation promoting factor from the surface of the tumour cell and its biological properties.

### MATERIALS AND METHODS

*Rat ascites hepatoma.*—Rat ascites hepatoma AH136B (Odashima, 1962) has been maintained in our laboratory by routine weekly passage of $1 \times 10^6$ AH136B cells injected i.p. into 80–100 g male rats of the Donryu strain. The majority of AH136B cells formed cell islands of varying size *in vivo*.

In vitro *assay for cell aggregation.*—This was performed essentially by a modification of the method of Moscona (1961*a*). One ml of the test sample at the same concentration (absorbancy 0·5 at 280 nm/ml) was mixed with 1 ml of cell suspension in a Falcon tube (1·5 × 9·5 cm) and incubated at 37°C in a roller tube culture apparatus, model Te-Her (Hirasawa Co., Tokyo, Japan) of one rotation/8 min. At intervals of 5, 15, 30 and 60 min after incubation, cell aggregation in both gross and microscopic features was recorded. The grading of the induced cell aggregation was achieved by counting the aggregating cells and floating cells respectively in the fluid at 30 min of incubation, at which macroscopic cell aggegation became satisfactory. The cell aggregates formed were carefully removed from the tube with a pipette, suspended in 2 ml of Hanks' balanced salt solution and dissociated mechanically by pipetting; total numbers of dissociated cells were counted by utilizing the microscopic apparatus with blood corpuscle counting chamber (Erma Optical Co., Tokyo, Japan). Total numbers of floating cells were also counted. Thus, the intensity of cell aggregation was roughly graded as follows: ++, over 70 ± 5% of originally suspended cells were aggregated; +, 50 ± 5% aggregated; ±, 30 ± 5% aggregated; and –, below 20% aggregated.

*Preparation of dissociated cell suspension.*—AH136B cell suspension was prepared as follows: the ascitic fluid was collected by i.p. puncture 10 days after transplantation of AH136B cells and diluted 1 : 10 with 0·45% NaCl solution. The cell suspension was kept at room temperature for 60 min to allow red blood cells to separate, and tumour cell islands were sedimented by centrifugation at 55 *g* for 10 min. After 3 washings with 0·45% NaCl, tumour cells islands were suspended in Hanks' balanced salt solution (free of calcium and magnesium) containing 0·1 mmol/l EDTA. After gentle shaking, the cells were mechanically dissociated by pipetting. The dissociated cells were sedimented by centrifugation at 55 *g* for 10 min and then washed with Hanks' balanced salt solution. Finally, the cell suspension containing $2 \times 10^5$ or $5 \times 10^5$ cells/ml was prepared in Hanks' balanced salt solution; most of the cells in the suspension were found to be free and the remaining cells (about 10%) were found in the form of small islands composed of 2–5 cells and did not interfere with evaluation of cell aggregation.

SV40-transformed cell suspension was prepared as follows: The cells in culture were generously supplied by Dr R. Mori, Department of Bacteriology, Kyushu University School of Medicine, Fukuoka, Japan. The cells were grown in Eagle's MEM (pH 7·4, Grand Island Biochemical Co., Grand Island, New York, U.S.A.) containing 10% calf serum and 5% tryptose phosphate broth (Difco Laboratories, Detroit, Michigan, U.S.A.) and collected after 7 days of cultivation. After washing with phosphate buffered saline, the cells were mechanically dissociated by pipetting and sedimented by centrifugation at 55 *g* for 10 min. The cell suspension was finally prepared with Hanks' balanced salt solution at concentration of $2 \times 10^5$ cells/ml; the majority of the cells in the suspension was found to be free.

Chang's cell suspension was prepared as follows: The cells were grown in Eagle's MEM containing 2% horse serum. The cells in 12-day-old culture were washed with Hank's balanced salt solution (free of calcium and magnesium), dissociated mechanically by pipetting and sedimented by centrifugation at 55 *g* for 10 min. The cell suspension was finally prepared with Hanks' balanced salt solution at concentrations of $5 \times 10^5$ or $2{\cdot}5 \times 10^6$ cells/ml; the majority of the cells in the suspension were found to be free.

Normal rat liver cell suspension was prepared as follows: liver cells of healthy male Donryu rats (80–100 g) were dissociated and collected according to the method of Anderson (1953). After perfusion with 20 ml of 0·1 mmol/l EDTA in physiological saline through the portal vein, the liver was excised and cut into small cubes or slices with a razor in 0·1 mmol/l EDTA solution. The cell suspension was filtered through 3 sheets of gauze and the liver cells were sedimented by centrifugation at 55 *g* for 10 min and washed. The cell suspension containing $5 \times 10^5$, $1 \times 10^6$ or $3 \times 10^6$ cells/ml was finally prepared with Hanks' balanced salt solution; most of the cells were found to be free. Red blood cells of healthy male Donryu rats (80–100 g) were collected and the cell suspension containing $5 \times 10^5$ or $1 \times 10^7$ cells/ml was prepared with Hanks' balanced salt solution.

*Chromatography*.—This was performed on columns of DEAE-Sephadex A-50 (3·5 mEq/g, Pharmacia, Uppsala, Sweden) prepared by the method of Porath and Lindner (1961) and Bio-gel A-5m (Bio-Road Laboratories, Richmond, California, U.S.A.) prepared by the method of Hjerten (1964). Protein concentrations were determined by the method of Warburg and Christian (1941) after measurement of the absorbancies at 280 nm and 260 nm of the test samples. Desired concentrations on protein solutions were obtained by vacuum pressure dialysis using the Visking cellulose tubing.

*Aggregation promoting factor in cell-free supernatant* (*Step* 1).—All procedures were performed in the cold (0°C). The ascitic fluid (200 ml), withdrawn 10 days after i.p. inoculation of AH136B cells, was diluted 1 : 10 with 0·45% NaCl and kept at room temperature for 60 min to allow red blood cells to separate. Tumour cell islands were collected by centrifugation at 55 *g* for 10 min, washed with 3 changes of 0·45% NaCl and resuspended in 200 ml of Hanks' balanced salt solution (free of calcium and magnesium). The cell suspension received 50 gentle pipettings and was allowed to stand for 3 h. The supernatant fluid, obtained after centrifugation at 300 *g* for 10 min, was further centrifuged at 10,000 *g* for 30 min for removing visible cellular components. Before assay the supernatant fluid was filtered through Millipore filters (pore size 0·3 μm). Tumour cells sedimented were stained with 0·5% trypan blue in Hanks' balanced salt solution; the numbers of damaged cells, as shown by diffuse staining with the dye, were only 2–5% of the suspended cells, indicating that the above procedures induced very little cell damage.

*Chromatography of aggregation promoting factor on DEAE-Sephadex* (*Step* 2).—All procedures were carried out in the cold (0°C). After dialysing against 0·02 mol/l phosphate buffer (pH 6·8) for 12 h, the supernatant fluid (10–20 ml, absorbancy 5–7 at 280 nm/ml) from Step 1 was applied to a column (2·0 × 20 cm) of DEAE-Sephadex equilibrated with 0·02 mol/l phosphate buffer (pH 6·8). Elution was accomplished by concentration stepwise changes of eluting buffers with the same pH (6·8) as follows: (1) 0·02 mol/l phosphate buffer; (2) 0·02 mol/l phosphate buffer plus 0·3 mol/l NaCl; (3) 0·02 mol/l phosphate buffer plus 0·5 mol/l NaCl; (4) 0·02 mol/l phosphate buffer plus 1·0 mol/l NaCl; and finally(5) 0·02 mol/l phosphate buffer plus 2·0 mol/l NaCl. The flow rate was 20 ml/h and 5 g effluent fractions were collected. Before use each effluent fraction was dialysed against Hanks' balanced salt solution for 12 h and then filtered through Millipore filters (pore size 0·3 μm).

*Gel filtration of aggregation promoting factor on Bio-gel* (*Step* 3).—All procedures were performed in the cold (0°C). The second peak (eluted in 0·02 mol/l phosphate buffer plus 0·3 mol/l NaCl on DEAE-Sephadex) from Step 2 was concentrated under vacuum pressure dialysis. The second peak (5 ml, absorbancy 4–6 at 280 nm/ml) was placed on a Bio-gel column (2·0 × 90 cm) equilibrated with Hanks' balanced salt solution. Filtration was performed at a rate of 5 drops/min and 4 g effluent fractions were collected. Before assay, each effluent fraction was dialysed against Hanks' balanced salt solution for 12 h and then filtered through Millipore filters (pore size 0·3 $\mu$m).

*Estimation of molecular size.*—According to the method of Andrews (1965), estimation of molecular size of an aggregation promoting factor was performed by gel filtration on a column (2·4 × 50 cm) of Sephadex G-200 (Pharmacia, Uppsala, Sweden) equilibrated with 0·05 mol/l Tris-HCl buffer (pH 7·4). The flow rate was 25 ml/h. Cytochrome *c* (12,400 mol. wt; Sigma, St Louis, Missouri, U.S.A.), bovine serum albumin (67,000 monomer mol. wt; 134,000 in dimer mol. wt; Armour, Kankakee, Illinois, U.S.A.), and urease (480,000 mol. wt; Merck, Darmstadt, Germany) were used as standard substances. The elution volumes for each substance were plotted against the logarithmic scale of molecular weight, and the linear relationship between the elution volume and logarithmic value of molecular weight was recorded.

### RESULTS

### *I. Isolation and partial purification of aggregation promoting factor*

(*a*) *Aggregation promoting factor in cell-free supernatant.*—Equal volumes (1 ml) of the cell-free supernatant fluid (absorbancy 0·5 at 280 nm/ml) from Step 1 and of AH136B cell suspension (5 × $10^5$ cells/ml) were mixed and incubated. Formation of macroscopic cell aggregates became visible as early as 5 min after the start of incubation. With increasing incubation time, the cell aggregates became larger, fused with each other and sedimented as a mass on the bottom of the culture tube (Fig. 1), the aggregated cells showing a tendency to arrange in a concentric pattern (Fig. 2). The activity of the factor (graded +) was found still present when assayed in 4-fold dilutions with Hank's balanced salt solution. On the other hand, AH136B cells were not aggregated in the absence of the supernatant fluid even after 2 h of incubation (Fig. 3). No difference in the potency of the supernatant fluid was revealed before and after dialysis against Hanks' balanced salt solution for 12 h, indicating that the active substance of the supernatant fluid was non-dialysable.

(*b*) *Aggregation promoting factor on DEAE-Sephadex.*—After elution of the cell-free supernatant (10–20 ml, absorbancy 5–7 at 280 nm/ml) from Step 1 on DEAE-Sephadex, 5 chromatographic peaks were obtained (Fig. 4) and the total yield, measured as absorbance at 280 nm, was about 70% of the proteins applied. The first peak contained 2·0%, the second 26·0%, the third 14·4%, the fourth 18·5% and the fifth 9·2%. However, the second peak (absorbancy 0·5 at 280 nm/ml) only caused a strong activity for AH136B cell aggregation under the same conditions as described above. Its affinity (graded +) was still present even when tested in 32-fold dilutions with Hanks' balanced salt solution, indicating an increase in the potency. No activity was revealed with the same concentration (absorbancy 0·5 at 280 nm/ml) of other peaks.

(*c*) *Aggregation promoting factor on Bio-gel.*—After elution of the second peak (5 ml, absorbancy 4–6 at 280 nm/ml) from Step 2 on Bio-gel, 2 chromatographic peaks were obtained (Fig. 5). The total yield, measured as absorbance at 280 nm, was about 95% of the proteins applied; the first peak contained 25% and the second 70%. The second peak (absorbancy 0·5 at 280 nm/ml) showed a strong activity for AH136B cell aggregation under the same conditions as described above; its activity (graded +) was still present even when tested in 42-fold dilutions with Hanks' balanced salt solution, indicating an increased potency. No activity was demonstrated with the first peak (absorbancy 0·5 at 280 nm/ml) under the same conditions. The potency of the aggregation promoting factor in each step of purification is summarized (Table I).

FIG. 1.—(a) Photograph of AH136B cell aggregate observed as a mass on the bottom of culture tube at 30 min of incubation after addition of an aggregation promoting factor in the cell-free supernatant fluid (Step 1); its potency was graded + +. (b) No AH136B cell aggregation was induced in the absence of the aggregation promoting factor. See text.

*II. Physico-chemical and biological properties of aggregation promoting factor*

(*a*) *Estimation of molecular size of aggregation promoting factor*.—The active substance from Step 3 was eluted on a Sephadex G-200 column. Elution volumes of the standard substances on this column were as follows: 260·0 ml for cytochrome *c*; 185·3 ml for bovine serum albumin monomer; 136·2 ml for bovine serum albumin dimer; and 177·7 ml for aggregation promoting factor. The linear relationship between the elution volume and logarithmic value of molecular weight was recorded and the molecular weight of this aggregation promoting factor was estimated at 72,000 ± 7200 (Fig. 6).

(*b*) *Effect of heat on aggregation promoting factor*.—The active substance from Step 3 was found to be thermostable because most of the potency of this substance remained unchanged when heated to to 60°C for 30 min. The activity of the substance was kept satisfactorily at 0°C or in the frozen state.

(*c*) *Effect of sugars on aggregation promoting factor*.—Sugar preparations such as alpha-methyl-D-glucopyranoside (Lot No. KK881, Nutritional Biochemical Co.,

FIG. 2.—Photomicrograph of aggregated AH136B cells at 30 min of incubation after addition of an aggregation promoting factor in the cell-free supernatant fluid (Step 1). See text. ×100.

FIG. 3.—Photomicrograph of dissociated AH136B cells in the absence of an aggregation promoting factor (Step 1) at 1·5 h of incubation. See text. ×100.

Fig. 4.—Chromatography of cell-free supernatant fluid (Step 1) on DEAE-Sephadex. Changes of the ionic strength of eluting buffers with the same pH (6·8) are indicated by arrows: (1) 0·02 mol/l phosphate buffer; (2) 0·02 mol/l phosphate buffer plus 0·3 mol/l NaCl; (3) 0·02 mol/l phosphate buffer plus 0·5 mol/l NaCl; (4) 0·02 mol/l phosphate buffer plus 1·0 mol/l NaCl; and (5) 0·02 mol/l phosphate buffer plus 2·0 mol/l NaCl. Effluent fractions were collected every 5 g. Each chromatographic peak (absorbancy 0·5 at 280 nm/ml) was diluted serially with Hanks' balanced salt solution, and tested for AH136B cell aggregation. See text.

Fig. 5.—Chromatography of the second peak (Step 2) on Bio-gel. The elution buffer was Hanks' balanced salt solution (pH 7·3). Effluent fractions were collected every 4 g. Each chromatographic peak (absorbancy 0·5 at 280 nm/ml) was diluted serially with Hanks' balanced salt solution and tested for AH136B cell aggregation. See text.

TABLE I.—*Purification of Aggregation Promoting Factor*

| Steps of purification | Total protein (mg) | Minimum effective dose (mg) |
|---|---|---|
| Factor in cell-free supernatant fluid* | 40·0 | 0·125 |
| Factor on DEAE-Sephadex | 4·0 | 0·016 |
| Factor on Bio-gel | 2·0 | 0·012 |

* Separated from 15 × $10^8$ AH136B cells.

† The minimum quantities of aggregation promoting factor necessary for inducing aggregation (graded +) of dissociated AH136B cells (5 × $10^5$/ml) was calculated according to the method of Warburg and Christian (1941). See text.

Cleveland, Ohio, U.S.A.), N-acetyl-D-glucosamine (Lot No. Lj6929, Nutritional Biochemical Co., Cleveland, Ohio, U.S.A.) and D-glucose (Lot No. IL4882, Wako Chemical Co., Osaka, Japan) were dissolved at concentrations of 100 mmol/l, 10 mmol/l, 1 mmol/l and 0·1 mmol/l in Hanks' balanced salt solution. Ten ml of AH136B cell suspension containing 5 × $10^5$ cells/ml were mixed with 10 ml of sugar solutions, and allowed to stand for 30 min at room temperature. To the mixture (2·0 ml), an equal volume of the active substance from Step 3 was added for assay of cell aggregation. The effect of the aggregation promoting factor was not, however, influenced in the presence of these sugar preparations even at highest concentration (100 mmol/l).

(*d*) *Reactions of aggregation promoting factor with various reagents.*—The active substance from Step 3 seemed to be a protein because positive results were obtained in the biuret, ninhydrin and Folin reactions. The reaction with phenol sulphate for sugar was also positive. On the other hand, the reactions with indol and orcinol for nucleic acid were all negative.

(*e*) *Effect of aggregation promoting factor on other cells.*—Following the same method as described above, the effect of the active substance (absorbancy 0·5 at 280 nm/ml) from Step 2 was assayed on SV40-transformed cells, Chang's cells, normal liver cells and red blood cells. The active substance was clearly effective in aggregating SV40-transformed cells (2 × $10^5$ cells/ml) and its potency was

FIG. 6.—Molecular weight determination of an aggregation promoting factor (Step 3) by gel filtration on Sephadex G-200. Each elution volume was plotted against the logarithmic scale of molecular weight. The elution volume of an aggregation promoting factor was approximately 177·7 ml. See text.

TABLE II.—*Comparison of Activities of Aggregation Promoting Factor from Rat Ascites Hepatoma Cells and of Concanavalin A on SV40-Transformed Cells*

| Materials tested | Duration of incubation (min) | Amounts of materials ($\mu$g) | | | | | | | | |
|---|---|---|---|---|---|---|---|---|---|---|
| | | 5·5 | 11 | 23 | 31 | 62 | 125 | 250 | 500 | 1000 |
| ConA* | 5 | − | − | − | − | − | − | ± | ± | + |
| | 15 | − | − | − | − | − | − | + | + | ++ |
| | 30 | − | − | − | − | − | − | + | + | ++ |
| APF† | 5 | − | ± | + | ++ | ++ | ++ | ++ | ++ | ++ |
| | 15 | − | ± | ++ | ++ | ++ | ++ | ++ | ++ | ++ |
| | 30 | − | + | ++ | ++ | ++ | ++ | ++ | ++ | ++ |

The cells at a concentration of $2 \times 10^5$/ml were scored for aggregation on a qualitative scale after 5, 15, 30 min of incubation at 37°C with appropriate concentrations of aggregation promoting factor (Step 2) and concanavalin A.

* Con A, Jack bean concanavalin A.

† APF, aggregation promoting factor (Step 2). See text.

apparently greater than that of Jack bean concanavalin A (Lot No. 110056, Calbiochem., San Diego, California, U.S.A.) (Table II). The action of the factor was less pronounced on Chang's cells; cell aggregation was not induced when assayed at concentration of $5 \times 10^5$ cells/ml, but became positive at the higher concentration of $2{\cdot}5 \times 10^6$ cells/ml. On the other hand, the substance was apparently ineffective for aggregating normal liver cells, even when assayed at higher concentration of $3 \times 10^6$ cells/ml. Similarly negative results were obtained with high concentrations ($5 \times 10^6$ or $1 \times 10^7$ cells/ml) of red blood cells.

(*f*) *Effect of aggregation promoting factor on casein and haemoglobin.*—Proteolytic activity of the active substance from Step 3 was tested by a modification (Hayashi *et al.*, 1962, 1965) of the casein digestion method of Kunitz (1947) and by a modification (Sliwinski, Doty and Landmann, 1959) of the haemoglobin digestion method of Anson (1939). The assay was performed at various pH ranges (3·0–6·0 against haemoglobin and 6·0–10·0 against casein) and concentrations (absorbancy 1·0–3·0 at 280 mn/ml) of the factor. It had no proteolytic activity.

### DISCUSSION

The observations described here demonstrated that a tumour cell aggregation promoting factor was released possibly from the cell surface from rat ascites hepatoma AH136B cells forming cell islands, when kept in cold Hanks' balanced salt solution (free of calcium and magnesium). It could be partially purified by chromatography using DEAE-Sephadex and by gel filtration using Bio-gel. The substance was assumed to be a thermostable glycoprotein with a molecular weight of about 72,000. This is of some interest in view of the well known evidence that sugar containing molecules of animal cell membranes may play a part in mediating cell adhesions, perhaps as " recognition sites ". The substance had no proteolytic activity when tested against casein and haemoglobin.

The action of this material was characterized by lack of inhibition of AH136B cell aggregation by sugar preparations which inhibit the effect of aggregation promoting factors such as Jack bean concanavalin A (Inbar and Sachs, 1969), wheat germ glycoprotein (Burger and Goldberg, 1967) and plant phytoagglutinins (Tomita *et al.*, 1970). This strongly suggests the presence of a binding site for the factor on the cell surface, different from the sites for aggregation promoting factors of plant origin mentioned above.

The factor also differed from concanavalin A in its higher aggregating activity when compared on SV40-transformed cells. The potency of 11 $\mu$g of the aggregation promoting factor seemed to correspond to that of 250 $\mu$g of Jack bean concanavalin A.

The substance was clearly effective for aggregating dissociated AH136B cells as well as SV40 transformed cells, but less effective for Chang' cells. On the other hand, it was apparently ineffective for normal liver cells and red blood cells of rats. This may be due to functional differences on the surface of hepatoma cells and liver cells which need further investigation.

The power to induce tumour cell aggregation has been seen in the serum (Tal, Dishon and Gross, 1964) and ascitic fluid of cancer patients (Mori, Akedo and Tanigaki, 1970) and of tumour bearing mice (Oppenheimer and Humphreys, 1971). However, the problem of whether such potency may be related to an aggregation promoting factor of cancer cell origin has not yet been established. We have recently demonstrated that a substance similar to the present aggregation promoting factor can be separated from the sera and ascitic fluid of rat ascites hepatoma AH136B or AH109A transplanted rats (Kudo and Hayashi, 1972) and this suggested that the factor was released from these cancer cells.

We would like to record our appreciation to Dr M. Koono for assay of proteolytic activity and to Dr M. Yoshinaga for discussion. This work was supported in part by special grants for cancer research from the Japanese Ministry of Education and by a grant from the Shionogi Pharmaceutical Company, Osaka, Japan.

### REFERENCES

ABERCROMBIE, M. & AMBROSE, E. J. (1962) The Surface Properties of Cancer Cells: A Review. *Cancer Res.*, **22**, 525.

ANDERSON, N. G. (1953) The Mass Isolation of Whole Cells from Rat Liver. *Science, N.Y.*, **117**, 627.

ANDREWS, P. (1965) The Gel Filtration Behaviour of Proteins Related to their Molecular Weights over a Wide Range. *Biochem. J.*, **96**, 595.

ANSON, M. L. (1939) The Estimation of Pepsin, Trypsin, Papain and Cathepsin with Hemoglobin. *J. gen. physiol.*, **22**, 79.

BURGER, M. M. & GOLDBERG, A. R. (1967) Identification of a Tumor-specific Determination on Neoplastic Cell Surfaces. *Proc. natn. Acad. Sci. U.S.A.*, **57**, 359.

COMAN, D. R. (1944) Decreased Mutual Adhesiveness, a Property of Cells from Squamous Cell Carcinomas. *Cancer Res.*, **4**, 625.

ENTERLINE, H. T. & COMAN, D. R. (1950) The Ameboid Motility of Human and Neoplastic Cells. *Cancer, N.Y.*, **3**, 1033.

HANES, F. M. & LAMBERT, R. R. (1912) Amöboid Bewegungen von Krebszellen als ein Factor des invasiven und metastatischen Wachstum maligner Tumoren. *Virchows Arch. path. Anat. Physiol.*, **209**, 12.

HAYASHI, H., MIYOSHI, H., NITTA, R. & UDAKA, K. (1962) Proteolytic Mechanism in Recurrence of Arthus-type Inflammation by Thiol Compounds. *Br. J. exp. Path.*, **43**, 564.

HAYASHI, H., UDAKA, K., MIYOSHI, H. & KUDO, S. (1965) Further Study of Correlative Behavior between Specific Protease and its Inhibitor in Cutaneous Arthus Reactions. *Lab. Invest.*, **14**, 665.

HAYASHI, H., YOSHIDA, K., OZAKI, T. & USHIJIMA, K. (1970) Chemotactic Factor Associated with Invasion of Cancer Cells. *Nature, Lond.*, **226**, 174.

HIRONO, I. (1958) Ameboid Motility of Ascites Hepatoma Cells and its Significance for their Invasiveness and Metastatic Speed. *Cancer Res.*, **18**, 1345.

HJERTEN, S. (1964) The Preparation of Agarose Spheres for Chromatography of Molecules and Particles. *Biochem. biophys. Acta*, **79**, 393.

HUMPHREYS, T. (1963) Chemical Dissolution and *in vitro* Reconstruction of Sponge Cell Adhesions. *Devl Biol.*, **8**, 27.

INBAR, M. & SACHS, L. (1969) Structural Difference in Sites on the Surface Membrane of Normal and Transformed Cells. *Nature, Lond.*, **223**, 710.

KATSUYA, H., KOONO, M. & HAYASHI, H. (1973) Separation of Tumor Cells by Activation of an Intrinsic Neutral Protease. In preparation.

KOONO, M., KATSUYA, H. & HAYASHI, H. (1974) Studies on the Mechanisms of Invasion in Cancer. IV. A Factor Associated with Release of Neutral Protease of Tumor Cell. *Int. J. Cancer*, **13**, 334.

KOONO, M., USHIJIMA, K. & HAYASHI, H. (1974) Studies on the Mechanisms of Invasion in Cancer. III. Purification of Neutral Protease of Rat Ascites Hepatoma Cell Associated with Production of Chemotactic Factor for Cancer Cells. *Int. J. Cancer*, **13**, 105.

KUDO, K. & HAYASHI, H. (1972) Aggregation-promoting Factor from Ascitic fluid and Serum of Tumor-bearing Rats and its Origin. *Proc. Jap. Cancer Ass.*, **31**, 80 (abstract).

KUNITZ, M. (1947) Crystalline Soybean Trypsin Inhibitor. II. General Properties. *J. gen. Physiol.*, **30**, 291.

LILIEN, J. E. (1968) Specific Enhancement of Cell Aggregation *in vitro*. *Devl Biol.*, **17**, 657.

MCCUTHEON, M., COMAN, D. R. & MOORE, F. B. (1948) Studies on Invasiveness of Cancer. Adhesiveness of Malignant Cells in Various Human Adenocarcinomas. *Cancer, N.Y.*, **1**, 460.

MORI, Y., AKEDO, H. & TANIGAKI, Y. (1970) Cell Aggregation Factor in AH-130 Hepatic Ascites Tumor Cell Fluid. *Proc. Jap. Cancer Ass.*, **29**, 143 (abstract).

MOSCONA, A. A. (1961*a*) Rotation-mediated Histogenetic Aggregation of Dissociated Cells. *Expl cell Res.*, **22**, 455.
MOSCONA, A. A. (1961*b*) Effect of Temperature on Adhesion to Glass and Histogenetic Cohesion of Dissociated Cells. *Nature, Lond.*, **190**, 408.
MOSKOWITZ, M. (1963) Aggregation of Cultured Mammalian Cells. *Nature, Lond.*, **200**, 854.
NAKAMURA, K. (1964) Transplantable Leukemia as an Ascites Tumor: its Histogenesis and Progress of the Disease. *J. natn. Cancer Inst. Monog.*, **16**, 149.
ODASHIMA, S. (1962) Comparative Studies on the Transplantability of Liver Cancers Induced in Rats Fed with 3′-methyl-4-dimethylamino-azobenzene for 3–6 months. *Gann*, **53**, 325.
OPPENHEIMER, S. B. & HUMPHREYS, T. (1971) Isolation of Specific Macromolecules Required for Adhesion of Mouse Tumour Cells. *Nature, Lond.*, **232**, 125.
OZAKI, T., YOSHIDA, K., USHIJIMA, K. & HAYASHI, H. (1971) Studies on the Mechanisms of Invasion in Cancer. II. *In vivo* Effects of a Factor Chemotactic for Cancer Cells. *Int. J. Cancer*, **7**, 93.
PORATH, J. & LINDNER, E. B. (1961) Separation Methods Based on Molecular Sieving and Ion Exclusion. *Nature, Lond.*, **191**, 69.
SATO, H. (1967) Cancer Metastasis: The Experimental Studies of Ascites Tumors. *Trans. Soc. Path. Jap.*, **56**, 9 (in Japanese).
SATO, H., SUZUKI, M. & KUROKAWA, U. (1966) Mechanism of Cancer Metastasis Formation. *Jap. J. clin. Path.*, **24**, suppl., 67 (in Japanese).
SATOH, H. (1967) Conditions Involved with Cancer Cell Proliferation. *Bull. Res. Inst. Tuberc. Leprosy (Sendai)*, **19**, 104 (in Japanese).
SLIWINSKI, R. A., DOTY, D. N. & LANDMANN, W. A. (1959) Over-all Assay and Partial Purification Procedures for Proteolytic Enzymes in Beef Muscle. *Agric. Fd Chem.*, **7**, 788.
TAL, C., DISHON, T. & GROSS, J. (1964) The Agglutination of Tumour Cells *in vitro* by Sera from Tumour Patients and Pregnant Woman. *Br. J. Cancer*, **18**, 111.
TOMITA, M., OSAWA, T., SAKURAI, Y. & UKITA, T. (1970) On the Surface Structure of Murine Ascites Tumors. I. Interactions with Various Phytoagglutinins. *Int. J. Cancer.*, **6**, 283.
WARBURG, O. & CHRISTIAN, W. (1941) Isolierung and Kristallisation des Gärungsferments Enolase. *Biochem. Z.*, **310**, 384.
WOOD, S. JR, ROBINSON, B. R. & MARZOCCHI, B. (1968) *In vivo* Studies of Tumor Behavior: Locomotion of and Interrelationships between Normal Cells and Cancer Cells. In *The proliferation and Spread of Neoplastic Cells*, (ed). The University of Texas, M. D. Anderson Hospital and Tumor Institute, Houston, Baltimore: Williams and Wilkins. p. 495.
YOSHIDA, K., OZAKI, T., USHIJIMA, K. & HAYASHI, H. (1970) Studies on the Mechanisms of Invasion in Cancer. I. Isolation and Purification of a Factor Chemotactic for Cancer Cells. *Int. J. Cancer*, **6**, 123.
ZEIDMAN, I. (1947) Chemical Factors in the Mutual Adhesiveness of Epithelial Cells. *Cancer Res.*, **7**, 386.


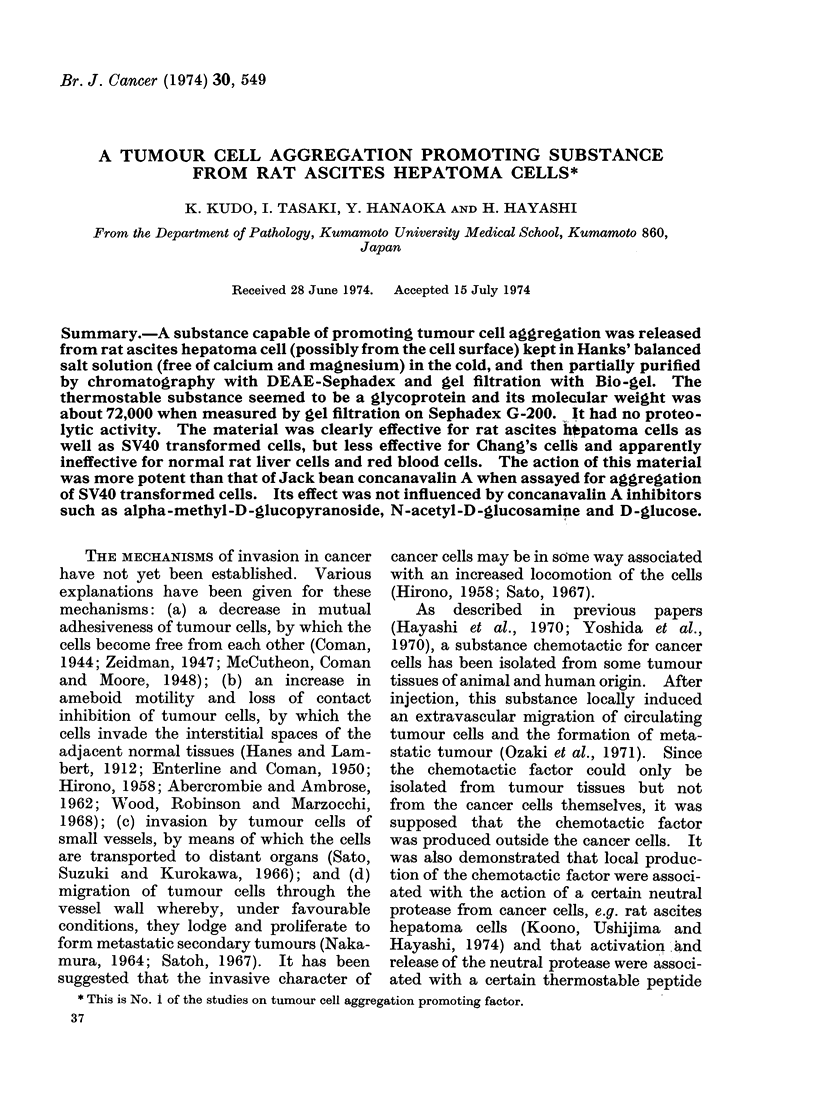

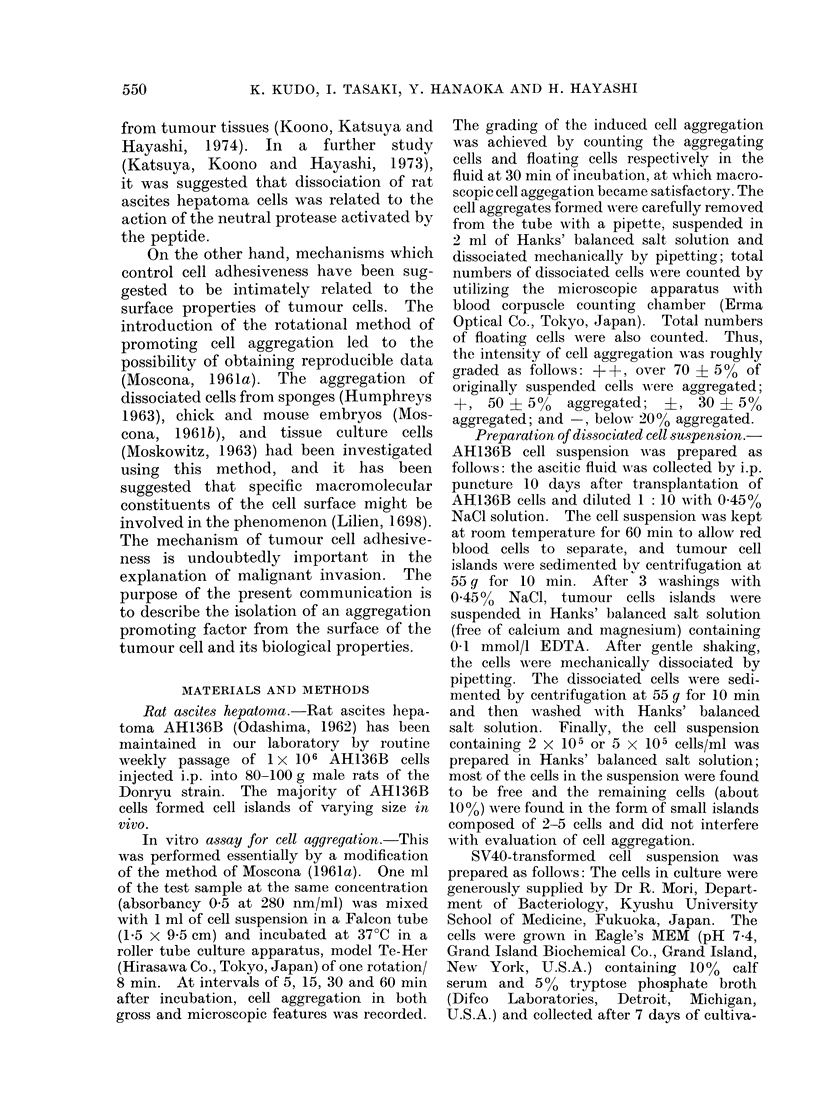

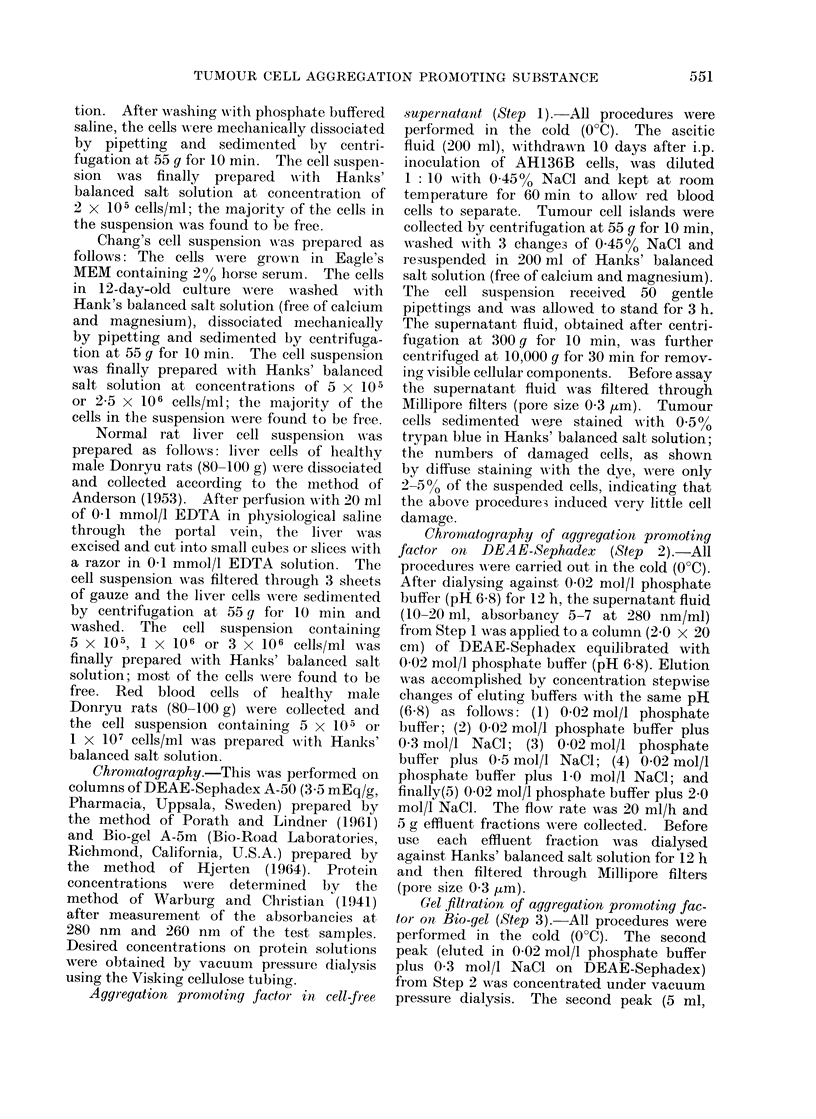

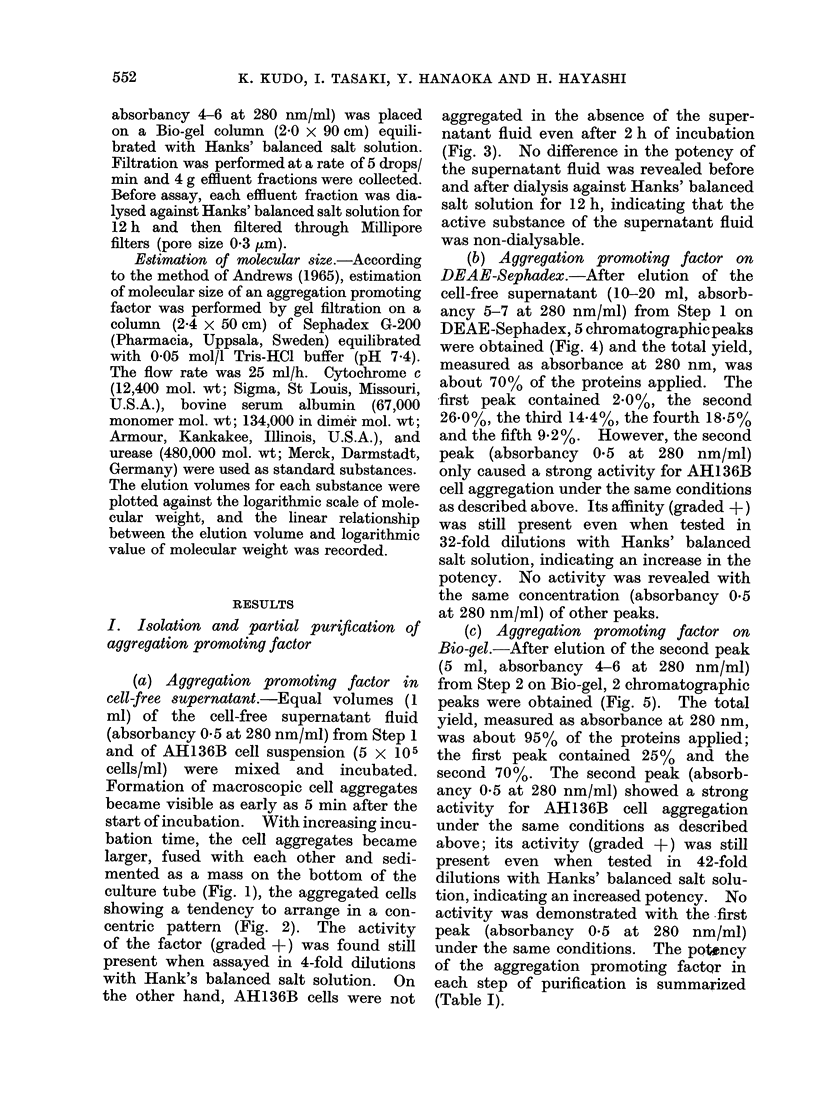

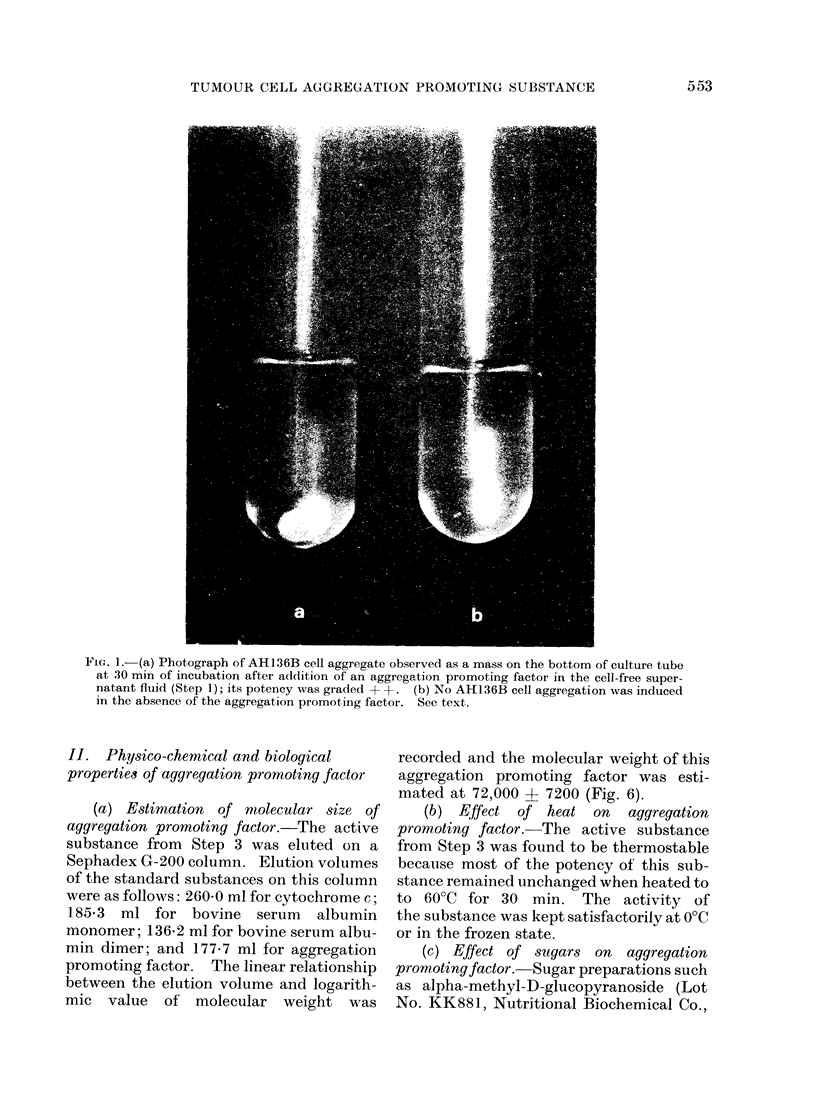

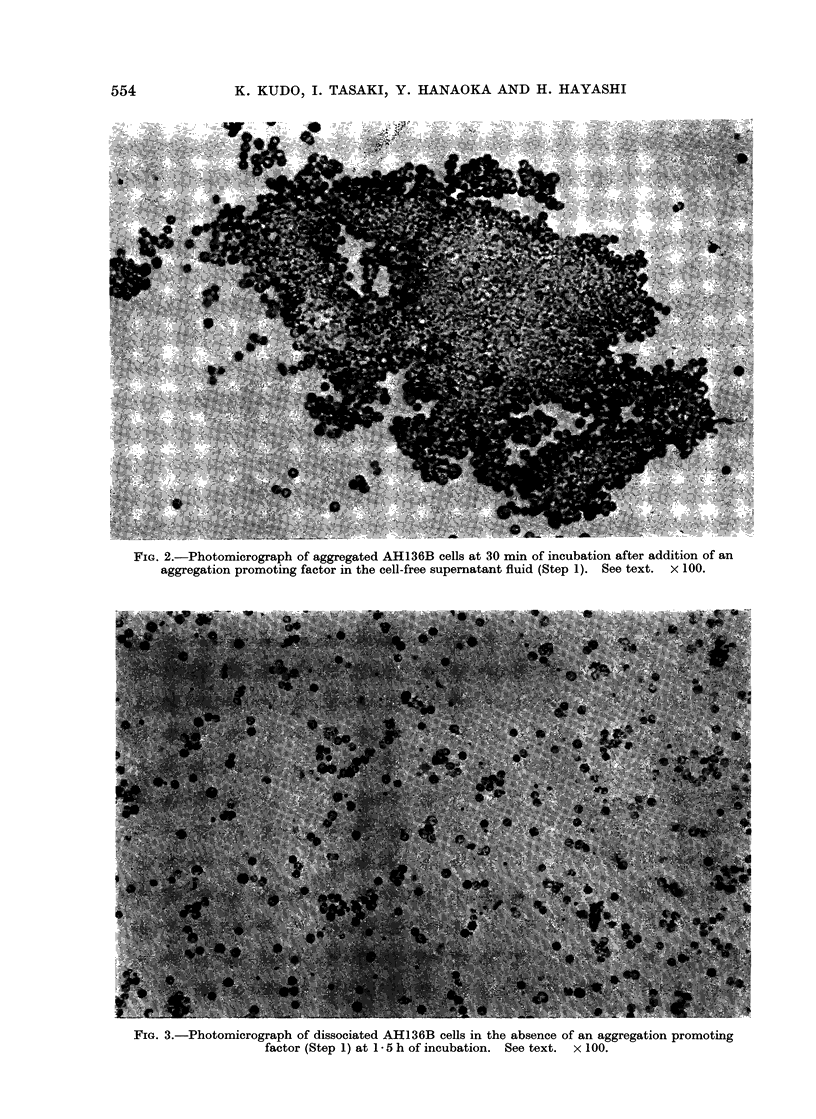

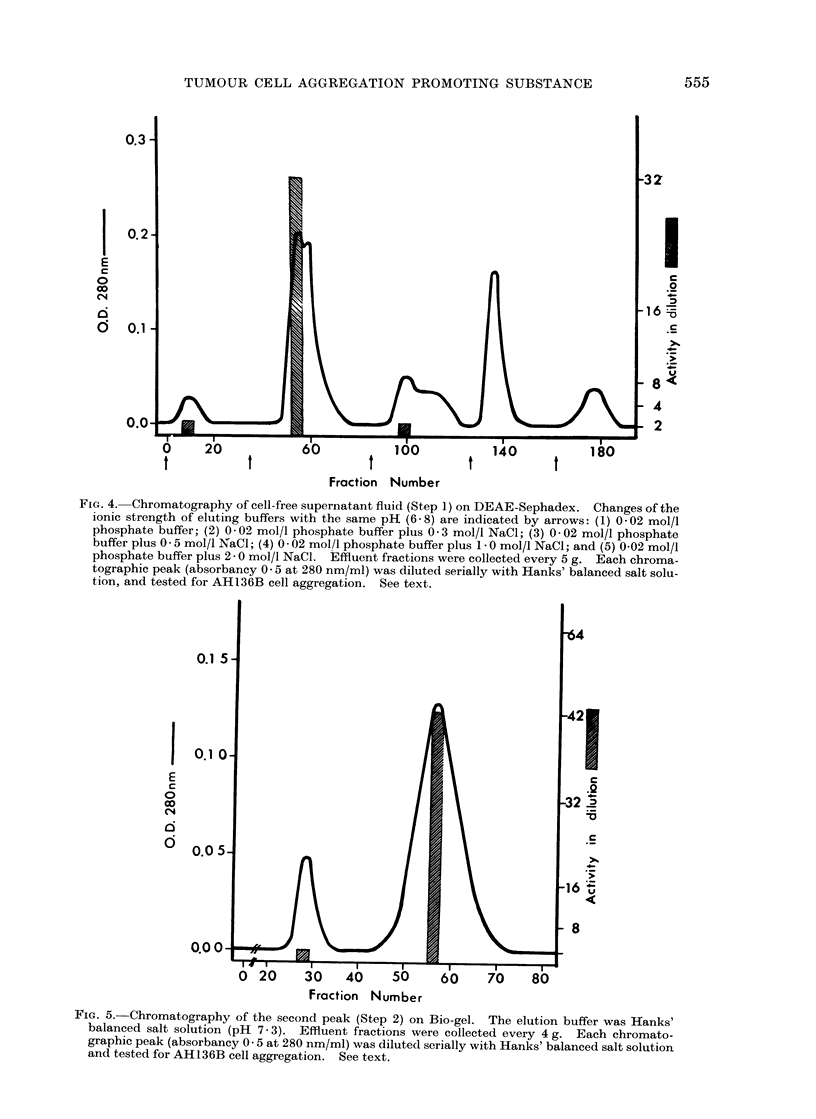

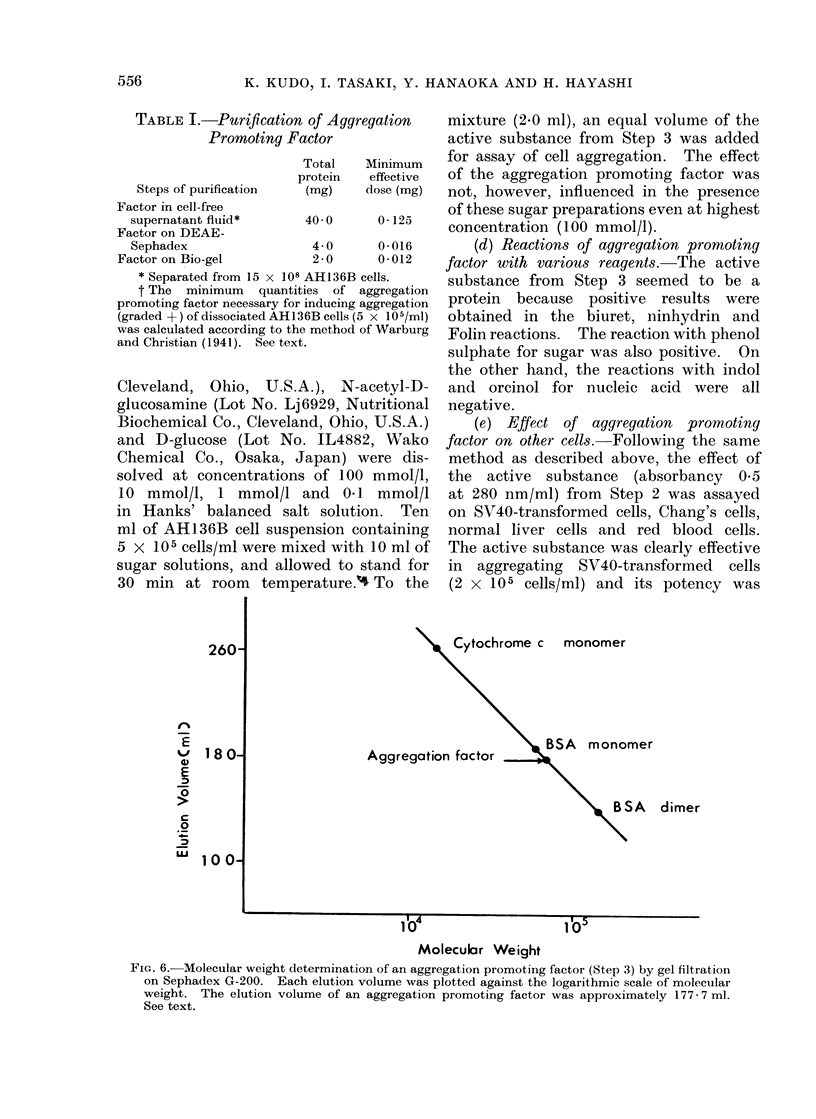

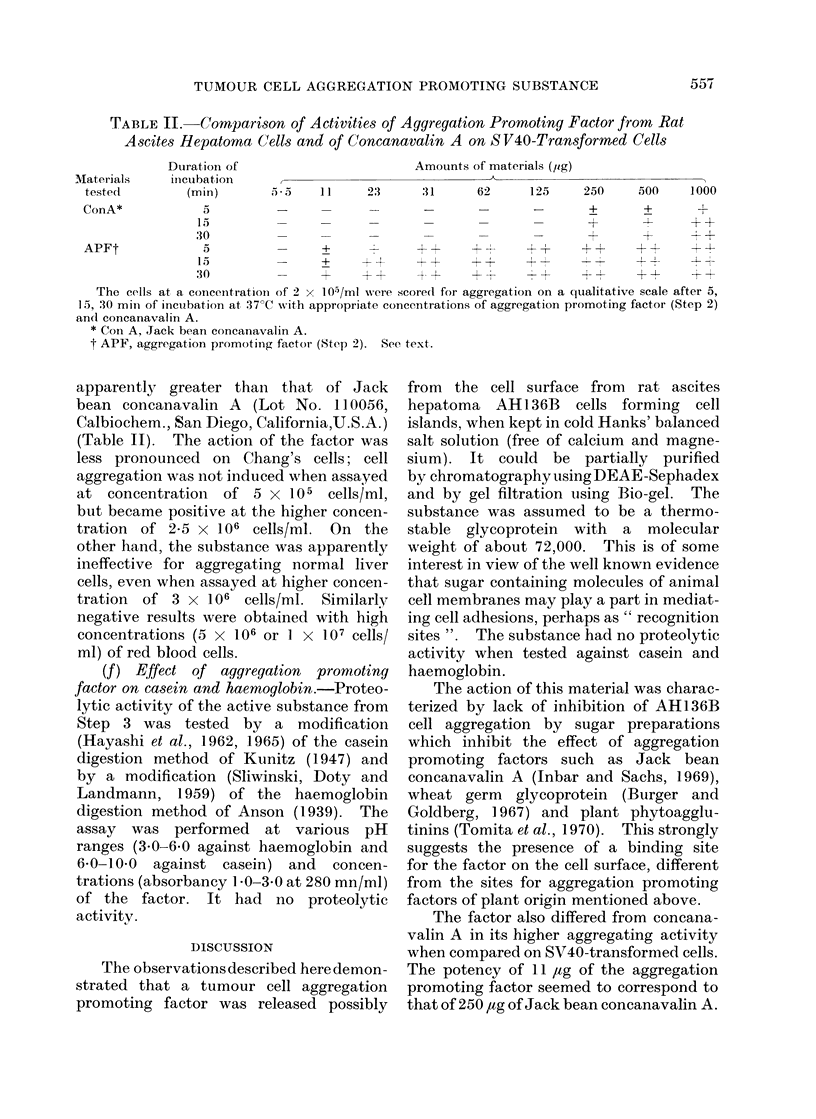

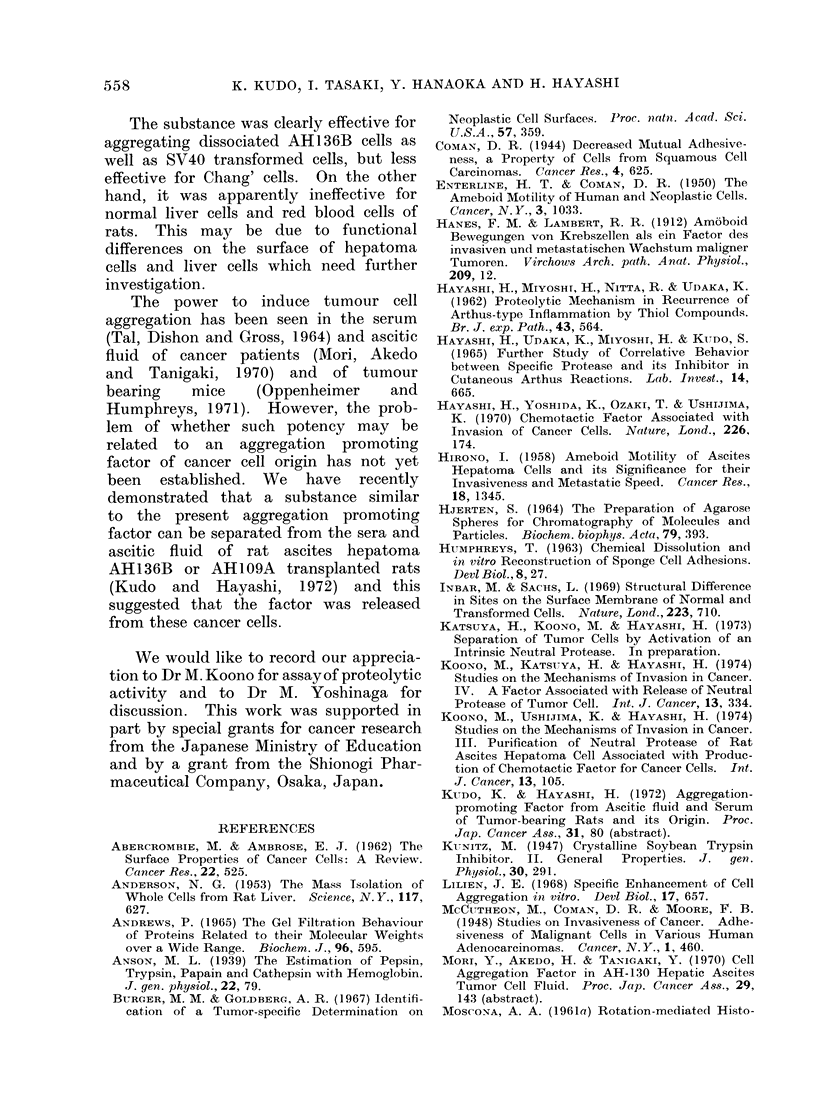

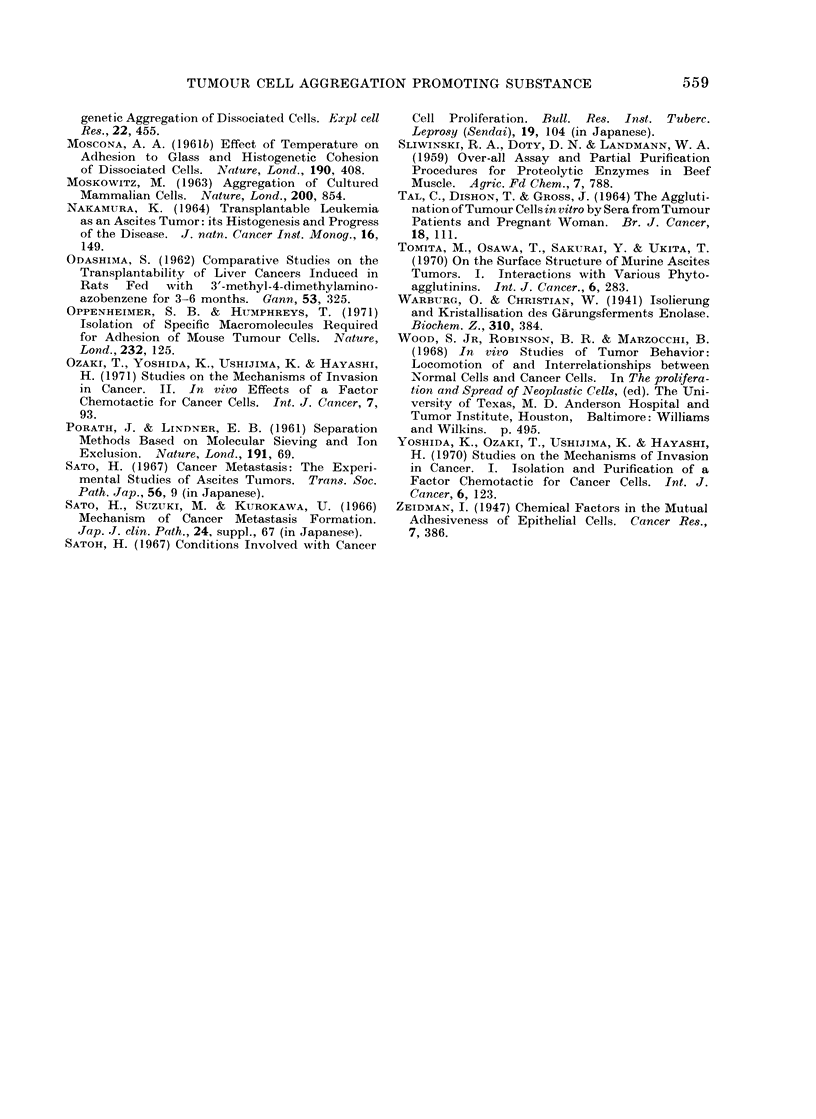


## References

[OCR_00849] ANDERSON N. G. (1953). The mass isolation of whole cells from rat liver.. Science.

[OCR_00854] Andrews P. (1965). The gel-filtration behaviour of proteins related to their molecular weights over a wide range.. Biochem J.

[OCR_00864] Burger M. M., Goldberg A. R. (1967). Identification of a tumor-specific determinant on neoplastic cell surfaces.. Proc Natl Acad Sci U S A.

[OCR_00876] ENTERLINE H. T., COMAN D. R. (1950). The ameboid motility of human and animal neoplastic cells.. Cancer.

[OCR_00894] HAYASHI H., UDAKA K., MIYOSHI H., KUDO S. (1965). FURTHER STUDY OF CORRELATIVE BEHAVIOR BETWEEN SPECIFIC PROTEASE AND ITS INHIBITOR IN CUTANEOUS ARTHUS REACTIONS.. Lab Invest.

[OCR_00907] HIRONO I. (1958). Ameboid motility of the ascites hepatoma cells and its significance for their invasiveness and metastatic spread.. Cancer Res.

[OCR_00913] HJERTEN S. (1964). THE PREPARATION OF AGAROSE SPHERES FOR CHROMATOGRAPHY OF MOLECULES AND PARTICLES.. Biochim Biophys Acta.

[OCR_00918] HUMPHREYS T. (1963). CHEMICAL DISSOLUTION AND IN VITRO RECONSTRUCTION OF SPONGE CELL ADHESIONS. I. ISOLATION AND FUNCTIONAL DEMONSTRATION OF THE COMPONENTS INVOLVED.. Dev Biol.

[OCR_00901] Hayashi H., Yoshida K., Ozaki T., Ushijima K. (1970). Chemotactic factor associated with invasion of cancer cells.. Nature.

[OCR_00923] Inbar M., Sachs L. (1969). Structural difference in sites on the surface membrane of normal and transformed cells.. Nature.

[OCR_00933] Koono M., Katsuya H., Hayashi H. (1974). Studies on the mechanisms of invasion in cancer. IV. A factor associated with release of neutral protease of tumor cell.. Int J Cancer.

[OCR_00938] Koono M., Ushijima K., Hayashi H. (1974). Studies on the mechanisms of invasion in cancer. 3. Purification of a neutral protease of rat ascites hepatoma cell associated with production of chemotactic factor for cancer cells.. Int J Cancer.

[OCR_00957] Lilien J. E. (1968). Specific enhancement of cell aggregation in vitro.. Dev Biol.

[OCR_00888] MAYASHI H., MIYOSHI H., NITTA R., UDAKA K. (1962). Proteolytic mechanism in recurrence of Arthus-type inflammation by thiol compounds.. Br J Exp Pathol.

[OCR_00980] MOSCONA A. (1961). Effect of temperature on adhesion to glass and histogenetic cohesion of dissociated cells.. Nature.

[OCR_00973] MOSCONA A. (1961). Rotation-mediated histogenetic aggregation of dissociated cells. A quantifiable approach to cell interactions in vitro.. Exp Cell Res.

[OCR_00985] MOSKOWITZ M. (1963). AGGREGATION OF CULTURED MAMMALIAN CELLS.. Nature.

[OCR_00995] ODASHIMA S. (1962). Comparative studies on the transplantability of liver cancers induced in rats fed with 3'-methyl-4-dimethylaminoazobenzene for 3-6 months.. Gan.

[OCR_01001] Oppenheimer S. B., Humphreys T. (1971). Isolation of specific macromolecules required for adhesion of mouse tumour cells.. Nature.

[OCR_01007] Ozaki T., Yoshida K., Ushijima K., Hayashi H. (1971). Studies on the mechanisms of invasion in cancer. II. In vivo effects of a factor chemotactic for cancer cells.. Int J Cancer.

[OCR_01014] PORATH J., LINDNER E. B. (1961). Separation methods based on molecular sieving and ion exclusion.. Nature.

[OCR_01040] TAL C., DISHON T., GROSS J. (1964). THE AGGLUTINATION OF TUMOUR CELLS IN VITRO BY SERA FROM TUMOUR PATIENTS AND PREGNANT WOMEN.. Br J Cancer.

[OCR_01046] Tomita M., Osawa T., Sakurai Y., Ukita T. (1970). On the surface structure of murine ascites tumors. I. Interactions with various phytoagglutinins.. Int J Cancer.

[OCR_01067] Yoshida K., Ozaki T., Ushijima K., Hayashi H. (1970). Studies on the mechanisms of invasion in cancer. I. Isolation and purification of a factor chemotactic for cancer cells.. Int J Cancer.

